# Teens with Type 1 Diabetes: How Does Their Nutrition Measure Up?

**DOI:** 10.1155/2018/5094569

**Published:** 2018-09-06

**Authors:** Eleanor Race Mackey, Lyndsay O'Brecht, Clarissa S. Holmes, Marni Jacobs, Randi Streisand

**Affiliations:** ^1^Children's National Health System, Psychology, Washington, DC, USA; ^2^University of Toronto, Toronto, Ontario, Canada; ^3^Virginia Commonwealth University, Pediatrics, Richmond, VA, USA; ^4^Georgetown University, Psychiatry, Washington, DC, USA

## Abstract

**Objective:**

To characterize the intake of macronutrient and fiber in adolescents with type 1 diabetes (T1D) and examine their association with health indicators.

**Methods:**

Baseline data from an RCT were examined. Adolescent-parent dyads (*n* = 257, mean age 12 ± 1.2 years, 49.4% girls) reported dietary intake via two separate 24-hour recall interviews during a two-week period. Demographic and medical variables were abstracted from questionnaires and medical charts.

**Results:**

Controlling for demographic and diet variables, a higher percentage of daily energy intake from fats was associated with poorer HbA1c. In contrast, an association between higher percent of energy intake from proteins and carbohydrates was found with higher systolic and diastolic BP, respectively.

**Conclusions:**

Many early adolescents with T1D did not meet diabetes nutritional guidelines. Lower adherence to nutritional guidelines, specifically more than recommended energy intake from fats, was associated with poorer HbA1c. Addressing nutritional guidelines and increasing adherence as part of treatment may improve health outcomes for youth with T1D.

## 1. Introduction

Type 1 diabetes (T1D), one of the most common childhood chronic illnesses, requires adherence to a complex daily routine for proper management [1, 2]. Poorer diabetes management and glycemic control increase risk for poorer outcomes, both concurrently and later in life [3]. However, adolescence is characterized by a period of poor adherence and glycemic control, in part due to the developmental task of establishing independence from caregivers, which often has negative consequences for diabetes management [4, 5].

Adhering to diabetes-specific guidelines for percentage of daily energy intake of macronutrients (i.e., carbohydrates, proteins, and fats), as well as intake of dietary fiber, is especially important for individuals with T1D to promote good ambient glycemic control and to prevent long-term consequences including cardiovascular disease [5–9]. Adolescents may choose to exert independence through their food choices, which, in turn, can be associated with the declines in glycemic control frequently found during this developmental period [1, 10]. Therefore, examination of the nutritional behaviors of adolescents with T1D and the connection between macronutrient and fiber intake and critical health indicators linked to diabetes-related complications and cardiovascular disease is important.

The dietary composition of adolescents with T1D is not likely to significantly differ from the general population, which is generally related to high fat consumption (e.g., [11]); however, the stakes are clearly higher for those with T1D due to the increased likelihood of poor health outcomes secondary to T1D. Additionally, research specifically comparing youth with and without diabetes found that youth with diabetes have higher fat intake [12]. Dietary research with adults who have type 1 diabetes also indicates difficulty adhering to nutrition guidelines (e.g., [13, 14]). Given the importance of dietary intake to glycemic control and to the health of youth with T1D, the nutritional content and behaviors of this population merit careful investigation.

Nutritional guidelines based on both the American Diabetes Association (ADA; [15]) and the International Society for Pediatric and Adolescent Diabetes (ISPAD; [7]) support using medical nutritional therapy (MNT) in order to optimize the metabolic profiles (i.e., lower LDL plasma levels) of individuals with diabetes and to offset risk factors for cardiovascular disease [4, 5, 9]. Specifically, MNT recommends provision of individualized nutrition plans as an essential component of disease management for type 1 diabetes. MNT can include counseling about carbohydrate counting, as well as specific recommendations for nutritional intake and associated behavioral strategies [15, 16]. Moreover, planning should take into account insulin regimen and personal and familial food preferences [7, 15]. For example, nutrition recommendations may vary based on insulin regimen. Conventional insulin therapy generally refers to a more structured insulin regimen in which youth have a set range of carbohydrate intakes per meal and fewer injections (e.g., 2–3 per day). Individuals on flexible/basal bolus therapy (administered via multiple injections or a pump) are able to use an insulin-to-carbohydrate ratio for food intake to tailor their insulin administration to what they choose to eat [9]. Flexible insulin regimens are associated with better glycemic control [17], yet they may inadvertently facilitate consumption of unhealthy foods and use of processed/packaged foods. The latter approach, in particular, may be appealing to the adolescent because it simplifies carbohydrate counting [18].

Few studies have explored specific macronutrient and fiber intake in adolescents with T1D. Initial evidence of suboptimal nutrition and its adverse relation to metabolic control has been demonstrated [19–23]. Although dietary intake is generally associated with concurrent glycemic control measured via continuous glucose monitoring [22], a direct intervention to improve dietary behaviors in youth did not result in improved glycemic control [24], suggesting that adherence to nutritional guidelines may be necessary for improved glycemic control, but not sufficient on its own. However, dietary behaviors also relate to other health indicators. For example, dietary intake, specifically low consumption of vegetables and fish, is related to retinal problems in youth with T1D [25]. Higher fiber intake is associated with reduced inflammation and lower mortality risk in adults with T1D [26, 27].

Given the association of dietary intake with health outcomes, the first aim of the current study is to describe the macronutrient and fiber intake of early adolescents with T1D. The second goal is to evaluate the association of macronutrient and fiber intake with important health indicators including glycemic control (hemoglobin A1c; HbA1c), obesity as indicated by body mass index, dyslipidemia as indexed by levels of plasma LDL, and hypertension as indexed by systolic and diastolic blood pressure (BP). It is hypothesized that only a small percentage of early adolescents with T1D will meet diabetes-specific nutritional guidelines and that macronutrient and fiber intake, as well as adherence to guidelines, will be associated with health indicators.

## 2. Methods

### 2.1. Participants

Early adolescents, aged 11 to 14, and their parents participated in a randomized controlled trial (RCT) at two mid-Atlantic children's hospitals. Participation in the RCT entailed completion of four brief sessions of behavioral intervention or diabetes education in conjunction with quarterly diabetes clinic visits over the course of 1 to 1 and 1/2 years. The intervention and outcomes are described in greater detail in a previously published paper [28]. The data for the current study were drawn from the baseline assessments of all of participants in the RCT with at least one completed dietary interview.

Eligibility requirements of the RCT included diabetes duration of at least 1 year, absence of severe complications (e.g., retinopathy and nephropathy) or other medical diagnoses (e.g., cancer and asthma), and English fluency. The sample consisted of 257 dyads composed of one adolescent with T1D (49% girls) and one parent (91% mothers). Mean age at baseline was 12.8 years (SD = 1.2), mean illness duration was 5.1 years (SD = 3.1), and mean HbA1c was 73 mmol/mol (SD = 4). Two-thirds (64%) of the samples were prescribed a flexible insulin regimen, i.e., ≥4 injections or basal/bolus injections, or continuous subcutaneous insulin infusion/pump. Participant characteristics are summarized in Table 1.

### 2.2. Procedures

Institutional review boards from each institution approved the larger study. Eligible families were identified from clinic lists and were mailed an informational letter. These families then received a follow-up telephone call from trained research assistants. At regularly scheduled diabetes clinic visit, each participant and his/her parent provided consent and assent, completed self-report questionnaires, and participated in separate parent and child 24-hour nutrition and diabetes self-care interviews. A second 24-hour retrospective dietary interview was completed over the telephone within two weeks of the baseline assessment. Completion of questionnaires took approximately 45 minutes, and each interview was approximately 15 minutes. Families received a $25 gift card in appreciation of their time. Of the 404 eligible families successfully contacted, 285 consented to participate (71%). Those who declined consent primarily cited lack of interest or time as the reason. Completed baseline data were provided by 257 parent-adolescent dyads (89%).

### 2.3. Measures

#### 2.3.1. Background Information

Demographic and medical information was obtained via questionnaire and a retrospective review of the medical charts. Socioeconomic status (SES) was calculated based on parental occupation and education [29], with higher scores related to lower SES. Coding was completed by research assistants. When there was not a clear category for a current career, the research assistant discussed with a senior study staff member and a joint decision was made. Insulin regimen was categorized as conventional (2–3 fixed injections/day) or flexible (≥4 injections, basal/bolus or pump) therapy. Parents reported the type of their child's nutrition plan (i.e., carbohydrate counting and exchanges) and the percentage of time their child adhered to his/her nutrition plan on average (i.e., ≤25% of the time, 26–50% of the time, 51–75% of the time, and >75% of the time). HbA1c and LDL cholesterol concentration were measured via blood assay at regular clinic visits and extracted from the medical record to represent metabolic control and lipid profile, respectively. HbA1c was assessed with the same measurement technology at each site (DCA 2000, 4.3–5.7%, Bayer Inc., Tarrytown, NY, USA). Height and weight also were measured at the baseline clinic visit, and body mass index (BMI) for age and gender percentile was calculated. Blood pressure (BP) also was measured and recorded.

#### 2.3.2. Nutrition

To assess macronutrient and fiber intake, youth and one parent separately completed the 24-hour diabetes interview (DI) [30]. The DI is a diary-like interview in which parents and adolescents separately describe the completion of diabetes self-care tasks and nutritional intake over the previous 24 hours. Parents and adolescents each completed the DI on the same day on two different occasions within a two-week span. The responses of each parent and youth report were combined for each interview using a formula created by the research team [31] and scored using the Food Processor® Nutrition Software (ESHA Research, Salem, OR, USA). A combination of the two reports via decision rules overcomes common parent-child discrepancies and reduces bias and source error [30]. To further increase reliability, an average score of the two interviews was analyzed. For the current analyses, percentage of energy intake composed of carbohydrates, proteins, and fats, as well as grams dietary fiber, was evaluated. The 24-hour dietary recall method is a reliable, valid, well-established measure of diabetes self-care behavior [32, 33], energy, and nutritional intake.

Parent- and youth-reported dietary intake was compared to current nutritional recommended guidelines (Table 2) for adolescents with T1D provided by the American Diabetes Association (ADA). The International Society for Pediatric and Adolescent (ISPAD) guidelines also were consulted as a secondary source when specific guidelines did not exist by the ADA [4, 9]. These guidelines differ from standard guidelines in that recommended ranges are similar, but narrower for adolescents with diabetes than in the general population (e.g., % of energy intake from carbohydrates is recommended at 45–65% for healthy adolescents and 50–55% for adolescents with diabetes) [34].

### 2.4. Data Analytic Plan

Analyses were conducted using SAS software, version 9.3 (SAS Institute Inc., Cary, NC). One of the 257 parent-adolescent dyads who completed baseline questionnaires was excluded from dietary analysis because they did not report dietary intake. To quantify the portion of the sample that met nutritional guidelines, each nutritional variable was recoded into dichotomous variables based on the cutoff recommended level for each nutrient (i.e., 0 = did not meet guidelines; 1 = met guidelines). Next, bivariate associations between dietary intake, demographic variables, metabolic control, blood pressure, and lipid profile were assessed with general linear regression. Nonlinear relationships between significant nutrition variables and health indicators also were explored by including exponential variables in linear models. Following bivariate analyses, in order to evaluate the overall and unique contributions of hypothesized predictors to outcomes, multivariable linear regression models were conducted by including demographic variables associated with a health indicator at *p* ≤ 0.10 in Step 1. Step 2 added nutrition variables associated with a health indicator at *p* ≤ 0.10 to Step 1 (model 2). Finally, a fully saturated model was tested that included demographic and nutrition variables associated with any health indicator at *p* ≤ 0.10 (model 3). Health indicators not associated with any nutrition variables were not included in the model testing. Continuous nutrition variables were given preference over categorized variables; however, significant categorized variables were explored in secondary models. Variables associated with health indicators at *p* ≤ 0.05 were considered significant in all models, and *R*^2^ was used to assess the amount of variance accounted for by the addition of nutrition variables to the model.

## 3. Results

### 3.1. Nutritional Recommendations

The proportion of participants who met nutritional recommendations by category and by type of regimen is summarized in Table 2. Only 25.8% of the samples met the guidelines for the percentage of their energy intake comprised of carbohydrates, 51.2% ate less than recommended, and 23.8% ate more than recommended. Only 45.9% of participants achieved recommendations for percentage of their energy intake from proteins, 44.7% ate less than recommended, and 9.3% ate more than recommended. Only 42% of youth met the guidelines for percent of energy intake as fats, and 51.6% of participants consumed more fats than recommended. Only 47.6% met the minimum guidelines for intake of dietary fiber. Participants on a flexible insulin regimen were significantly more likely to meet dietary fiber guidelines as compared to those on a conventional regimen (*χ*^2^(1) = 5.23, *p* = 0.02).

### 3.2. Adherence

Approximately half of parents (51.7%) reported that their child followed their nutrition plan less than 75% of the time. Using 75% of the time as a cutoff, parents of youth on a flexible regimen were significantly more likely to report that their child followed their nutrition plan the majority of time than those on a conventional regimen (*χ*^2^(1) = 13.65, *p* < 0.001).

### 3.3. Health Recommendations

Targets for HbA1c were met more frequently by those participants on a flexible regimen versus conventional regimen (*χ*^2^(1) = 4.03, *p* = 0.05). Of the health outcomes evaluated, only 21.3% achieved an HbA1c below 58 (mmol/mol) and was within the glycemic goal target range. Consideration of weight revealed 64.8% had a BMI percentile within the normal range, although 21.4% were classified as overweight and 13.6% had obesity. Moreover, 64.5% of participants had measured LDL cholesterol levels less than 100 mg/dL (2.59 mmol/L). Approximately 90% had measured blood pressure below 130/80 mmHg. These health indicators did not differ significantly by type of insulin regimen (*χ*^2^ all *p* > 0.05).

### 3.4. Bivariate Associations

Bivariate associations between demographic and nutrition variables and all health indicators are presented in Table 3. Demographic associations were quite varied, with longer duration of disease associated with higher systolic BP (*β* = 0.46, *p* = 0.04) and higher LDL (*β* = 1.53, *p* = 0.03). Lower SES category was related to poorer HbA1c (*β* = 0.61, *p* < 0.001), as was a conventional insulin regimen (*β* = 0.69, *p* = 0.002). Non-Caucasian ethnicity and older age were each associated with every poorer health indicator except BMI percentile, which was not statistically significantly related to either non-Caucasian ethnicity or age. A higher percentage of energy intake from proteins was the only nutrient associated with higher systolic BP (*β* = 0.42, *p* = 0.01), whereas a higher percentage of energy intake from carbohydrates was significantly associated with higher diastolic BP (*β* = 0.13, *p* = 0.04). The majority of nutritional variables were associated with HbA1c including fats (*β* = 0.05, *p* < 0.001), carbohydrates (*β* = −0.05, *p* < 0.001), and dietary fiber (*β* = −0.04, *p* = 0.02). No significant nutrition associations were noted for BMI percentile or LDL, and no evidence of nonlinear associations were seen.

### 3.5. Regressions Predicting Health Indicators

Results of regression analyses are shown in Table 4. Overall, in Step 1, demographic variables accounted for 9% of the variance in systolic BP, with older age (*β* = 2.09, *p* < 0.001) and longer diabetes duration (*β* = 0.43, *p* < 0.05) significantly contributing to the model predicting systolic BP. The addition of nutritional variables in Step 2 increased the variance explained by 2% points. Specifically, controlling for relevant demographic factors, a higher percentage of energy intake from proteins was significantly associated with higher systolic BP (*β* = 0.36, *p* = 0.03). Although nutritional variables were not significantly associated with diastolic BP controlling for relevant demographic factors, inclusion of nutritional variables marginally improved *R*^2^ (6% vs. 4%), showing contribution to the variance in diastolic BP. In Step 3, a higher percentage of energy intake from carbohydrates was associated with higher diastolic BP (*β* = 0.27, *p* < 0.05) The health indicator most highly associated with study variables was HbA1c, as 20% of the variance in outcome was accounted for by the model including relevant demographic and nutrition variables. A higher percentage of energy intake from fats was associated with higher HbA1c controlling for all other variables in the model (*β* = 0.05, *p* = 0.02). Fully saturated models did not alter these conclusions or improve percentage of variance explained.

## 4. Discussion

The current study reveals that many primarily middle-class early adolescents with T1D do not meet national nutritional guidelines set by the American Diabetes Association [4]. Nevertheless, most health indicators are relatively favorable and within normal limits at this age, despite suboptimal glycemic control. Perhaps more importantly, the current study provides evidence that adherence to nutritional guidelines for macronutrient intake is associated with better glycemic control, consistent with prior research. Additionally, this study demonstrates a link between higher percentage of energy intake from fats and poorer glycemic control in a young adolescent T1D sample, above and beyond the contribution of demographic and other dietary variables. Finally, longer diabetes duration was related to higher systolic blood pressure and higher LDL in the current pediatric sample, consistent with the increased risk of hyperlipidemia in diabetes [35, 36].

A higher percentage of energy intake from fats was related to poorer glycemic control after accounting for other study demographic, disease, and nutritional factors. Taken together, demographic variables and diet variables explained 20% of the variance in glycemic control, consistent with existing T1D research [22, 37]. In contrast, a higher percentage of energy intake from proteins was associated with higher systolic BP and a higher percentage of energy intake from carbohydrates related to higher diastolic BP. In the current sample, nutritional intake was not associated with BMI percentile or LDL cholesterol. Further, insulin regimen, conventional versus flexible, did not distinguish groups on the basis of nutritional intake or health indicators, with the exception of HbA1c, for which a higher percentage of participants on a flexible regimen met the guidelines. These findings are slightly different from previous research [37] that found variation in nutrition and health status by insulin regimen.

The current study illustrates that a sizable majority of youth did not meet ADA or ISPAD nutritional guidelines and may be at risk for cardiovascular disease and other health complications later in life. Early inklings of this risk are found in the present association between longer disease duration and higher LDL cholesterol and higher systolic blood pressure. Diabetes carries a known morbidity for hyperlipidemia, and therefore, youth may benefit from close monitoring of health status and macronutrient intake, particularly saturated fats, to prevent onset of cardiovascular disease [38]. Parents were aware that their adolescents were not following their nutritional plan, as over half (51%) reported that their adolescent followed their specific nutrition plan less than 75% of the time. However, parents of youth on flexible regimens were more likely to report that their adolescents were following nutrition guidelines. In support of our findings, existing studies have reported that adolescents with T1D do not meet the recommended ADA guidelines [39, 40].

Our study adds to the literature by finding an association between dietary intake and health indicators of glycemic control and blood pressure in early adolescents. That is, specific dietary components in the present study had a direct relation to established health indicators, beyond the contribution of demographic factors and general nutritional status which were controlled statistically. Previous work has examined the association between nutrition and health status in Chinese adults with T1D, whose diets likely are dissimilar to those of American adolescents [41]. Nevertheless, links were found among dietary patterns with HbA1c and LDL cholesterol in this Asian sample. Work in youth with T1D indicates that carbohydrate intake, when examined alone, is associated with lipid profiles, BMI, and HbA1c [20] and saturated fat intake is associated with HbA1c [19]. The current study not only supports these findings but also adds new information through examination of multiple demographic and nutritional variables simultaneously, regardless of insulin regimen. Adolescents and their parents may perceive that improved insulin delivery and dosing systems allow more freedom in food choices, but these data indicate that a heart-healthy diet has ramifications beyond traditional short- and long-term cardiac status and, in fact, directly relates to level of glycemic control.

A novel direct association also was found between macronutrient consumption and BP, although BP was not elevated overall in the current sample. Specifically, consumption of a higher percentage of energy from proteins related to higher systolic BP and a higher percentage of energy intake from carbohydrates related to diastolic BP. Importantly, evidence of dietary links to glycemic control and BP status based on just two samples of nutritional intake from two days is compelling and requires further study. More subtle associations may be found with more extensive sampling and other methods of assessing dietary intake. With an average disease duration in the present sample of just five years, the importance of understanding the dietary correlates of glycemic and cardiac status is underscored by the fact that longer diabetes duration was associated with higher LDL levels, a known risk factor for cardiovascular disease [42].

Consistent with previous literature [43, 44], Table 3 shows that demographic variables were associated with nutrition behaviors and diabetes health indicators. Specifically, older age places youth at risk for poorer health indicators overall. However, age was not associated with patterns of nutritional intake. Longer disease duration was associated with higher systolic BP and LDL. Non-Caucasian adolescents with T1D reported similar nutritional intake to Caucasians but were more likely to have overall poorer health indicators, suggesting that factors other than nutritional behaviors may place minority youth at greater risk for cardiovascular disease. Consistent with previous literature, low SES was associated with higher HbA1c values (e.g., [45]). Differences in disease-related variables, such as duration of illness and insulin regimen, may place early adolescents at different levels of risk for a confluence of poorer nutritional behaviors and health indicators. For example, longer duration of illness was associated with a higher systolic BP leading to a potential risk of hypertension and was associated with higher LDL, which potentiates for dyslipidemia. Adolescents on flexible regimens may have higher intake of carbohydrates and lower intake of proteins than peers on conventional regimens, which could be important for dieticians counseling adolescents on flexible regimens regarding the importance of adhering to nutrition guidelines.

## 5. Clinical Implications

When youth with T1D are first diagnosed, especially as younger children, their care and management fall solely to their parents. At the time of diagnosis, most families receive “survival skills” training, which incorporates nutrition guidelines and medical nutrition therapy. However, early adolescence represents a period of increased independence and self-management, as well as greater freedom in food choices. Early adolescents may not be routinely included in initial diabetes education such that they may be unaware of the importance of balanced nutrition to manage their HbA1c and to prevent future health risk. The present study's findings underscore the ADA recommendation of yearly meetings with a registered dietician such that nutrition counseling is a routine and frequent part of diabetes education and management. Particularly pressing is the need to help early adolescents understand the importance of adherence to nutrition recommendations to optimize their glycemic control and to decrease their risk for future cardiovascular disease [5, 9].

Given that education alone is not always sufficient to produce changes in behavior, for optimal efficacy, nutrition counseling could include focus on individual and cultural preferences, motivation, and self-efficacy to make healthful choices, as well as familial financial considerations. When adolescents and families have difficulty adhering to nutritional guidelines, clinical psychologists may be a critical component of care in order to promote adherence. Integration of technology may be an appealing facet of diabetes care to engage adolescents and promote self-monitoring behaviors as a means to support healthful decision-making. Technology may represent a fruitful avenue to keep nutritional education and reeducation updated and enjoyable.

## 6. Limitations

The current study is strengthened by the use of multimethod and multisource data at multiple time points. However, the cross-sectional nature of the study makes it impossible to determine casual relations among demographic, nutritional, and health variables. Moreover, the number of analyses increases risk for type 1 error. Future research should examine these relationships longitudinally, particularly as adolescents reach young adulthood and beyond. Moreover, although parents and youth reported their nutritional intake at two different time points using the gold standard for assessment of dietary intake, self-report of food intake is often underreported [32] and may not accurately represent actual consumption. Future research should incorporate other methods, such as direct observation or innovative technologies such as remote food photography method [46]. Other factors that affect glycemic control, such as meal timing, physical activity, and insulin dosing, should be simultaneously evaluated. Although the current sample is representative of the two institutions from which it was drawn, it may not be representative of all youth with type 1 diabetes.

## 7. Conclusions

Overall, many early adolescents with T1D did not meet guidelines for nutritional intake. Nonadherence to nutritional guidelines, above and beyond the influence of demographic variables, was associated with poorer HbA1c and places youth at risk for later health complications. Regular nutritional education, particularly for early adolescents, along with behavioral adherence to the guidelines may prove crucial for maintenance of better glycemic control and prevention of future cardiovascular disease.

## Figures and Tables

**Table 1:** Demographic and baseline characteristics.

	Mean (SD) or %	Range
*Demographic*		
Youth age (years)	12.8 (1.2)	11–15.3
Youth sex, % girls	49.4	
Youth ethnicity, % Caucasian	69.5	
Hollingshead SES, % level 1 or 2	53.8	
*Medical*		
Diabetes duration (years)	5.1 (3.1)	1–13.6
Insulin regimen		
2–3 injections per day (%)	35.3	
Basal/bolus (≥4 shots per day, %)	20.4	
CSII insulin pump (%)	44.3	
*Nutrition intake*		
% energy from carbohydrates (DI)	49.6 (8.3)	19.8–74.0
% energy from fats (DI)	35.3 (7.4)	12.8–55.0
% energy from proteins (DI)	15.9 (4.0)	6.7–29.8
Dietary fiber (g/1000 kcal (g/4184 kJ), DI)	14.7 (6.3)	3.8–38.3
*Health indicators*		
HbA1c (mmol/mol)	73 (4)	37–130
Systolic blood pressure (mmHg)	113.8 (10.6)	85–148
Diastolic blood pressure (mmHg)	64.0 (8.1)	44–94
LDL cholesterol (mg/dL)	89.3 (28.9)	6.0–187.0
BMI percentile	69.4 (24.5)	3–99

Note: SES = socioeconomic status; DI = dietary interview; BMI=body mass index.

**Table 2:** Frequency of participants meeting nutritional recommendations.

	Recommendation	Insulin regimen	
		Conventional (%)	Flexible (%)
*Nutritional intake*			
% energy from carbohydrates	50–55^1^	24.7	26.6
% energy from proteins	15–20^2^	48.8	44.7
% energy from fats	<35^1^	35.6	46.1
Cholesterol (mg/day)	<200^2^	54.4	57.6
Dietary fiber (g/1000 kcal (g/4184 kJ))	14^1^	37.8	52.9^∗^
*Health indicators*			
HbA1c (mmol/mol)	≤58^2^	14.1	25.2^∗^
BMI (normal, %)	5th–85th^2^	58.9	68.5
Systolic BP (mmHg)	<130/80 (or <90th %)^2^	93.0	91.9
Diastolic BP (mmHg)	<130/80 (or <90th %)^2^	97.7	96.9
LDL cholesterol (mg/dL (mmol/L))	<100 (2.6)^2^	62.5	52.1

^1^Recommendation from the International Society for Pediatric and Adolescent Diabetes. ^2^Recommendation from the American Diabetes Association. ^∗^Values significantly different between regimens.

**Table 3:** Bivariate associations among demographic and nutritional variables and all health measures.

	Systolic BP		Diastolic BP		HbA1C		BMI%		LDL	
	*β*	*p* value	*β*	*p* value	*β*	*p* value	*β*	*p* value	*β*	*p* value
Age	**2.18**	**<0.0001**	**0.92**	**0.03**	**0.17**	**0.05**	0.63	0.62	**4.46**	**0.007**
Sex (boys vs. girls)^1^	0.47	0.73	−0.72	0.49	0.09	0.68	−4.72	0.13	−2.20	0.59
Duration	**0.46**	**0.04**	0.18	0.31	0.003	0.82	−0.15	0.77	**1.53**	**0.03**
SES	1.17	0.14	0.68	0.27	**0.61**	**<0.0001**	1.86	0.32	2.13	0.37
Ethnicity (non-Caucasian vs. Caucasian)^2^	**2.96**	**0.04**	**2.78**	**0.01**	**0.84**	**0.0003**	5.53	0.10	**8.77**	**0.04**
Insulin regimen^3^	−0.85	0.55	1.41	0.19	**0.69**	**0.002**	5.29	0.10	3.82	0.37
Fats (% energy)	0.0001	0.99	−0.14	0.06	**0.05**	**0.0002**	−0.02	0.92	−0.28	0.33
Carbohydrates (% energy)	−0.05	0.51	**0.13**	**0.04**	**−0.05**	**0.0004**	0.10	0.58	0.07	0.78
Proteins (% energy)	**0.42**	**0.01**	0.14	0.28	0.05	0.07	0.31	0.42	0.78	0.12
Dietary fiber	−0.09	0.42	−0.12	0.13	**−0.04**	**0.02**	−0.21	0.39	−0.38	0.22
Fats (in range versus)										
Less than recommended	1.14	0.69	0.20	0.93	−0.24	0.60	−2.21	0.74	12.46	0.12
More than recommended	1.86	0.19	–0.60	0.58	**0.70**	**0.001**	1.35	0.67	−0.52	0.90
Carbohydrates (in range versus)										
Less than recommended	−0.41	0.80	−0.08	0.95	**0.74**	**0.003**	−2.21	0.56	−3.76	0.44
More than recommended	−1.43	0.46	1.29	0.38	0.01	0.97	−4.43	0.32	−0.88	0.88
Proteins (in range versus)										
Not in range	1.17	0.39	−1.12	0.28	0.21	0.34	−4.91	0.11	2.78	0.50
Dietary fiber (in range versus)										
Not in range	−2.22	0.64	**−7.07**	**0.05**	0.61	0.42	−14.89	0.18	4.19	0.75

Estimates based on linear regression analysis. Bold = *p* < 0.05. ^1^Boys coded as 0. ^2^Caucasian coded as 1. ^3^Flexible regimen coded as 1.

**Table 4:** Multivariable associations between demographic and nutritional variables and nutrition-associated health measures.

*R* ^2^	Systolic BP			Diastolic BP			HbA1C		
	Model 1	Model 2	Model 3	Model 1	Model 2	Model 3	Model 1	Model 2	Model 3
	0.09	0.11	0.12	0.04	0.06	0.08	0.14	0.20	0.21
	*β*	*β*	*β*	*β*	*β*	*β*	*β*	*β*	*β*
*Demographic variables*									
Age	2.14^‡^	2.09^‡^	2.05^‡^	0.92^∗^	0.92^∗^	0.66	0.19^∗^	0.17^∗^	0.17^∗^
Duration	0.42^∗^	0.43^∗^	0.37	—	—	0.11	—	—	0.009
SES^2^	—	—	1.32	—	—	0.26	0.50^‡^	0.44^‡^	0.46^‡^
Ethnicity^3^	2.94^∗^	2.51	2.39	2.77^†^	2.89^†^	1.85	0.36	0.44	0.42
Insulin regimen^4^	—	—	−2.40	—	—	1.03	0.35	0.26	0.26
*Nutrition variables*									
Fats (% energy intake)	—	—	0.11	—	−0.03	0.09	—	0.05^∗^^,5^	0.05^∗^
Carbohydrates (% energy intake)	—	—	0.11	—	0.11	0.27^∗^	—	0.005^5^	0.004
Proteins (% energy intake)	—	0.36^∗^	0.47^∗^	—	—	0.31	—	0.04	0.04
Dietary fiber	—	—	−0.00	—	—	−0.12	—	−0.02	−0.02

^1^Estimates based on linear regression analysis. Model 1 includes demographic variables significant at *p* < 0.10 in bivariate models. Model 2 includes variables in model 1 + nutrition variables significant at *p* < 0.10 in bivariate models. Model 3 is a fully saturated model that includes all variables significant for any outcome in bivariate models. ^2^SES: categorical with lower categories indicating higher SES. ^3^Non-Caucasian coded as 0 vs. Caucasian coded as 1. ^4^Conventional coded as 0 vs. flexible coded as 1. ^5^Categorical “not in range” or “less/more than recommended” as 0 vs. “in range” as 1. ^∗^*p* < 0.05; ^†^*p* ≤ 0.01; ^‡^*p* ≤ 0.001.

## Data Availability

The data used to support the findings of this study are available from the corresponding author upon request.
